# Improved electroretinographic responses following dietary intervention in a patient with Refsum disease

**DOI:** 10.1002/jmd2.12147

**Published:** 2020-07-12

**Authors:** Matthew D. Benson, Ian M. MacDonald, Melissa Sheehan, Shailly Jain

**Affiliations:** ^1^ Department of Ophthalmology and Visual Sciences University of Alberta Edmonton Alberta Canada; ^2^ Nutrition Services, Alberta Health Services Stollery Children's Hospital Edmonton Alberta Canada; ^3^ Department of Medical Genetics University of Alberta Edmonton Alberta Canada

**Keywords:** electroretinography, phytanic acid, Refsum disease, retinal degeneration

## Abstract

Refsum disease is a rare inherited metabolic disorder arising from a defect in peroxisomal metabolism. Patients lack the functional enzyme phytanoyl‐CoA hydroxylase, resulting in perturbed alpha oxidation of fatty acids. Phytanic acid accumulates in nervous and adipose tissue and leads to several disease phenotypes including early‐onset retinal degeneration, hearing loss, peripheral neuropathy, anosmia, and cerebellar ataxia, among others. Currently, restricting dietary phytanic acid is the only means of altering the chronic sequelae and the disease course. While dietary intervention has been demonstrated to improve peripheral neuropathy, ichthyosis, and ataxia, there have been no reports of improved retinal function in patients with Refsum disease. We describe the case of a 51‐year‐old patient with molecularly and biochemically confirmed Refsum disease who underwent electroretinography before and after beginning a phytanic acid‐restricted diet. His post‐intervention 30 Hz flicker electroretinogram demonstrated significantly improved waveform amplitudes and implicit times, suggesting improved retinal function. Thus, we propose that the possibility exists for some visual recovery in these patients and we highlight the utility of performing standardized electroretinography to assess treatment response in Refsum disease.

**Synopsis**

Retinal function may improve in patients with Refsum disease who are managed with a diet to reduce phytanic acid levels.

## 1 INTRODUCTION

Refsum disease is a rare autosomal recessive disorder that is primarily caused by mutations in *PHYH*, a gene that encodes the peroxisomal enzyme phytanoyl‐CoA hydroxylase. This enzyme catalyzes the alpha‐oxidation reaction of a 3‐methyl branched‐chain fatty acid known as phytanic acid. The accumulation of phytanic acid in nervous tissue and adipose tissue results in the constellation of clinical findings that characterize the disorder. Patients universally present with an early‐onset retinal dystrophy that leads to significant visual impairment in childhood. Sensorineural hearing loss, ichthyosis, anosmia, peripheral sensorimotor neuropathy, cerebellar ataxia, short metacarpals and metatarsals, and cardiac arrhythmias occur in this disease, but all of these findings are not typically present in every case.^1^

If not promptly recognized and treated, Refsum disease can be associated with significant morbidity and even mortality. A UK study that included 15 patients with Refsum disease identified retinitis pigmentosa in nearly every patient by the age of 40.^2^ Fortunately, dietary restriction of phytanic acid reduces morbidity and can slow the progression of and even reverse signs of peripheral neuropathy, ataxia, and ichthyosis.^1^, ^3^ While there are no reports of improved retinal function with dietary intervention, there is evidence to support a substantially slower rate of retinal degeneration with preserved vision into adulthood.^1^, ^3^

We present a case of molecularly confirmed Refsum disease in a 51‐year‐old patient who had previously been clinically diagnosed with nonsyndromic retinitis pigmentosa. We highlight the long interval between initial symptom onset and final diagnosis to illustrate that nonocular symptoms are not always readily apparent but should be ascertained in any patient with a retinal dystrophy, especially if occurring at a young age. Finally, we compare the results of electroretinography before and after dietary intervention and propose that retinal function may indeed improve with phytanic acid restriction.

## 2 CASE REPORT

A 51‐year‐old male from Barbados was referred to the Ocular Genetics clinic in April 2018 with a clinical diagnosis of retinitis pigmentosa that had been made 10 years prior. He described a long‐standing history of nyctalopia that began when he was 9 to10 years old that was accompanied by reduced peripheral vision. He felt that his visual symptoms had been slowly worsening over time. A review of symptoms was positive for hearing loss that had started 4 years ago and seemed to be gradually progressing. In addition, he had chronic dry skin and a recent onset of intermittent paresthesias of the upper and lower extremities. He denied difficulties with balance or loss of smell.

His past medical history was significant for non‐insulin‐dependent diabetes mellitus, hypercholesterolemia, and erectile dysfunction. His current medications included candesartan, amlodipine, rosuvastatin, low‐dose aspirin, and metformin. He had no history of smoking and he had no known medication allergies. There was no significant family history of ocular disease or of symptoms that could be suggestive of Refsum disease.

**FIGURE 1:**
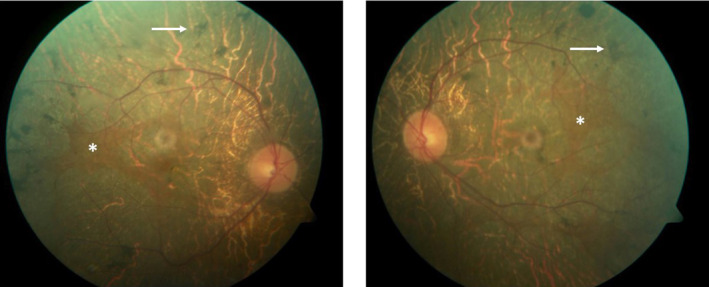
Color fundus photos of the right and left eye demonstrating bilateral diffuse retinal pigment epithelial (RPE) degeneration, mid‐peripheral pigment clumps (arrow), and retinal arteriolar narrowing. There are small scalloped regions of preserved RPE centrally in both eyes (asterisk)

When examined, his spectacle‐corrected Snellen visual acuity was 20/80 + 2 OD and 20/50 + 2 OS, not improving with pinhole correction. His refractive error was −2.75 + 2.00 × 135 OD and − 1.50 + 1.25 × 043 OS with a +2.50 reading add OU. His pupils were equal and reactive to light and accommodation with no relative afferent pupillary defect. A slit‐lamp examination of the anterior segment revealed mild posterior subcapsular lens changes OD and the presence of anterior vitreous cells OU. Posterior segment examination demonstrated a significant retinopathy OU with diffuse retinal pigment epithelial (RPE) degeneration, mid‐peripheral pigment clumps, and retinal arteriolar narrowing (Figure 1). The optic discs appeared grossly normal without any significant signs of pallor. There were no appreciable signs of diabetic retinopathy.

**FIGURE 2:**
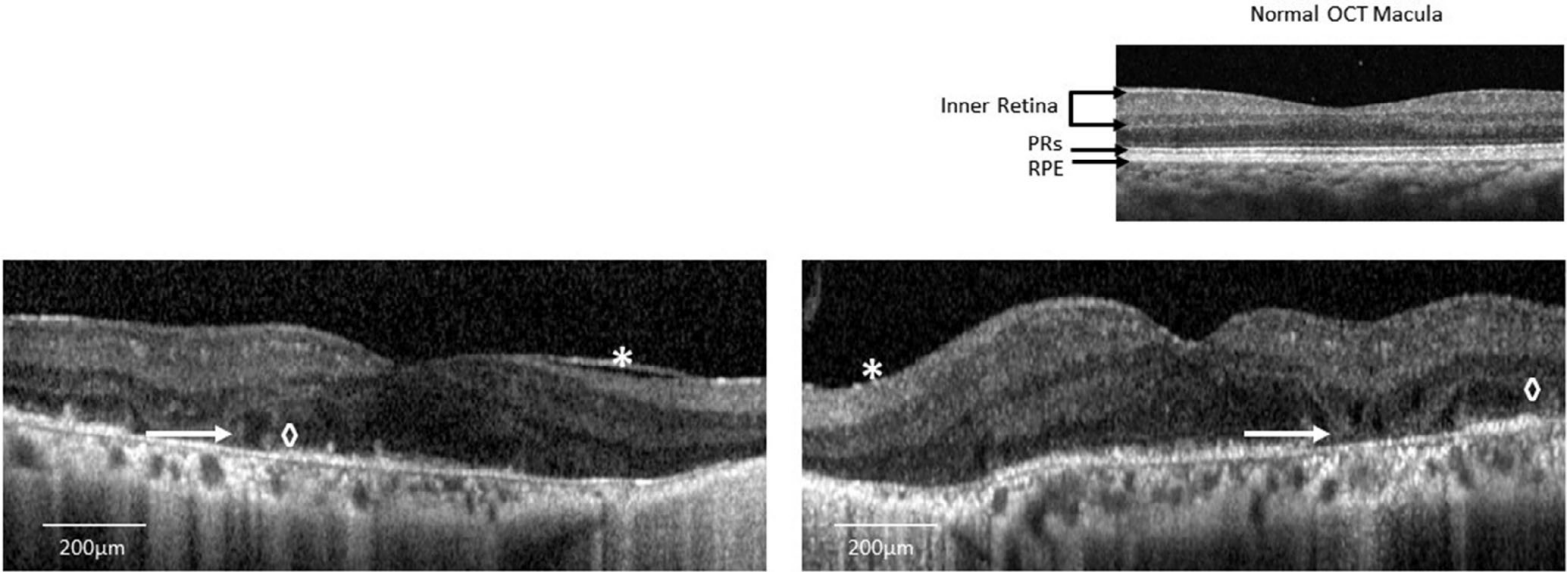
Spectral domain optical coherence tomography (OCT) of the right and left eyes demonstrating extensive outer retinal degeneration with attenuation of the outer nuclear layer, ellipsoid layer (photoreceptors), and RPE (arrow). Scattered outer retinal tubulations are visible in both eyes (diamond) along with bilateral epiretinal membranes (asterisk). Normal OCT of the macula given for reference (PR, photoreceptor layer; RPE, retinal pigment epithelial layer)

Spectral domain optical coherence tomography (OCT) revealed extensive outer retinal degeneration with attenuation of the outer nuclear layer, ellipsoid zone, and RPE. Scattered outer retinal tubulations were present along with bilateral epiretinal membranes (Figure 2). Blue‐peak fundus autofluorescence demonstrated small regions of foveal increased autofluorescence demarcated by scalloped borders. The remainder of the retina had decreased autofluorescence, in keeping with extensive RPE degeneration. An octopus visual field test confirmed significant peripheral field constriction with approximately 30° of residual field in either eye when interrogated with the V4e target.

**FIGURE 3:**
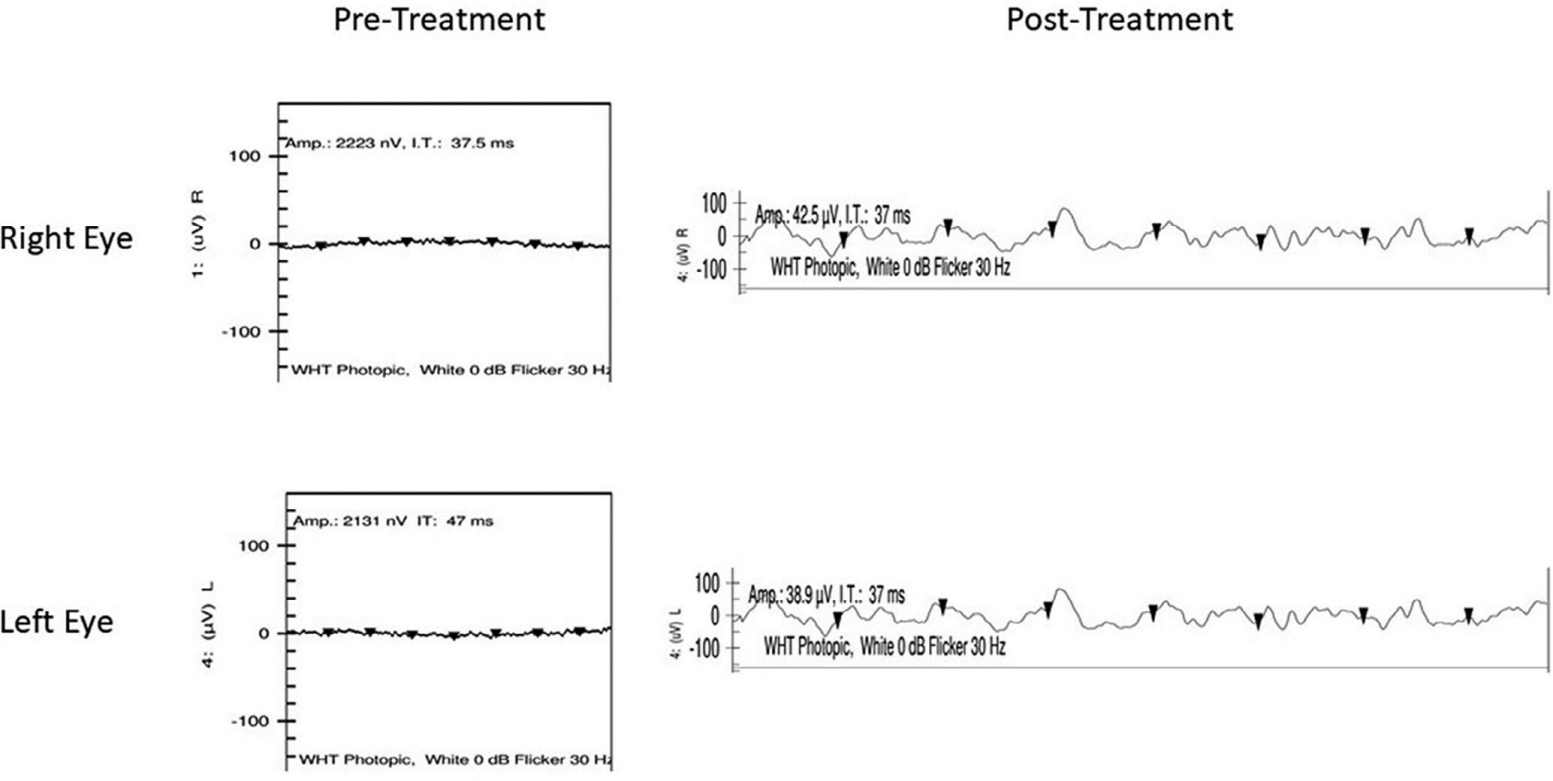
Pre‐ and post‐dietary intervention 30 Hz flicker electroretinograms of the right and left eye performed with DTL electrodes and conforming with the standardized ISCEV protocol. Waveform amplitudes increased from 2.2 μV to 42.5 μV (normal 68‐118 μV) in the right eye and increased from 2.1 μV to 38.9 μV (normal 68‐118 μV) in the left eye post‐intervention. Implicit times were reduced from 47 ms to 37 ms (normal 25‐29.8 ms) in the left eye but were largely unchanged in the right eye. Arrowheads represent each 30 Hz flicker stimulus and resulting waveform (*x*‐axis = time [ms]; *y*‐axis = voltage [μV])

A full field electroretinogram (ffERG) had been performed in 2015 using DTL electrodes with the International Society for Clinical Electrophysiology of Vision (ISCEV) standard parameters. The rod‐driven responses and cone‐driven bright flash were extinguished. The 30 Hz flicker response was severely diminished with an amplitude of 2.2 μV (normal 68‐118 μV) and implicit time of 37.5 ms (normal 25‐29.8 ms) OD and an amplitude of 2.1 μV (normal 68‐118 μV) and implicit time of 47.0 ms (normal 25‐29.8 ms) OS (Figure 3).

Given the early‐onset retinal degeneration coupled with the recent development of nonocular symptoms, a molecular diagnosis was sought and a 110‐gene retinitis pigmentosa molecular genetic panel was ordered (Blueprint Genetics, Helsinki, Finland). The patient was found to have homozygous frameshift variants in *PHYH* (c.57_67dup [p.Gly23Alafs*8]). Combining the patient's clinical and genetic results, a diagnosis of Refsum disease was provided. Subsequently, the patient was referred to Medical Genetics for further evaluation.

Initial serum phytanic acid levels were elevated at 449 μmol/L (normal <30 μmol/L). Baseline pristanic acid levels were 0.12 μmol/L (normal <1.5 μmol/L). Baseline fasting glucose was 5.6 mmol/L (normal <6.1 mmol/L) and hemoglobin A1C was 6.1% (normal <6.2%). Dietary restriction of phytanic acid was implemented with the assistance of a metabolic dietitian. The main resource used was “refsumdisease.org” and a handout was created to help our patient follow a “low” phytanic acid diet (Table S1). The handout was reviewed by other Canadian centers who follow patients with Refsum disease. Prior to initiating diet therapy, a diet history, including a 24‐hour recall, showed that the patient regularly consumed high phytanic acid foods including beef, fish and homogenized milk. Phytol, a precursor of phytanic acid, is released as a component of chlorophyll during the fermentation of green plants in ruminants. Therefore, high phytanic acid foods are mainly those derived from ruminant animals such as cow, sheep, goat, and high fat dairy products.^4^ In addition to avoiding of such high phytanic acid foods, other recommendations were to limit fasting to 8 to 10 hours, avoid rapid weight loss and implement a strict illness management plan to provide 120% of estimated energy requirements as well as decrease maximum fasting to 4 to 6 hours.

A cardiology assessment confirmed the absence of arrhythmias or other related cardiac disease. In addition, a neurology evaluation, which included a brain MRI, was unremarkable aside from impaired peripheral proprioception detected by physical examination. In summary, the patient demonstrated typical clinical features of Refsum disease including a retinal dystrophy, hearing loss, ichthyosis, and peripheral neuropathy but notably lacked anosmia, ataxia, shortened metacarpals and metatarsals, and cardiomyopathy.

In follow‐up 4 months later, the patient's serum phytanic acid was effectively reduced to 273 μmol/L (normal <30 μmol/L). The patient reported strictly following the dietary recommendations as listed (Table S1) prior to this level but did not provide a 3‐day food record and a 24‐hour dietary recall was vague. As such, phytanic acid consumption could not be quantified. Other than the dietary modifications, the patient did not have other interventions that could explain the decrease in phytanic acid level (he did not have lipid plasmapheresis or apheresis). His fasting glucose was slightly higher than previous at 7.3 mmol/L (normal <6.1 mmol/L) and his hemoglobin A1C was slightly elevated at 6.3% (normal <6.2%). He described improved energy with fewer symptoms of pins and needles in his upper and lower extremities. Interestingly, he felt that his vision may have improved following implementation of the low phytanic acid diet. As a result, we elected to repeat his 30 Hz flicker ERG as this was the only recordable electrophysiologic response prior to intervention. Furthermore, we felt that the ERG would be the most objective indicator of any change in retinal function. Repeat 30 Hz flicker ERG testing was performed in September 2019, using DTL electrodes and followed the same protocol as the previous ERG in 2015. The amplitude of the waveform was 38.9 μV (normal 68‐118 μV) OD with a 37 ms (normal 25‐29.8 ms) implicit time and 42.5 μV (normal 68‐118 μV) OS with a 37 ms (normal 25‐29.8 ms) implicit time, suggesting a functional improvement in cone‐driven responses (Figure 3). Surprisingly, his Snellen visual acuity measured poorer at 20/150 + 2 OD and 20/70 + 2 OS; however, he was able to obtain 20/60 OU which was not substantially different than when he had first presented. Regular follow‐up with Ophthalmology, Medical Genetics, and Cardiology was arranged to monitor the control of his disease.

A repeat serum phytanic acid level was performed 3 months later and measured as 396 μmol/L (normal <30 μmol/L). Although higher than the previous reading, this was still lower than his baseline level. Importantly, the patient admitted to being noncompliant with the dietary recommendations for the 2 to 3 months prior to this level and was fasting for 13 hours regularly in the time frame prior to this level. A 3‐day food record was requested but not provided by the patient.

## 3 DISCUSSION

Refsum disease is a rare multisystem disorder caused by the accumulation of phytanic acid due to deficiency in the peroxisomal enzyme phytanoyl‐CoA hydroxylase. The precise disease mechanism has yet to be determined; however, accumulation of phytanic acid has been shown to disrupt calcium homeostasis, increase oxidative stress, and decouple ATP synthesis in mitochondria, ultimately leading to cell apoptosis.^5^

The onset of symptoms in Refsum disease can range from 7 months of age to 50 years of age.^1^ Typically, however, the initial presenting symptoms begin in the second decade of life and are ophthalmic in nature and include nyctalopia, constricted visual fields, and eventual reduced central visual acuity. Thus, this disorder often presents first to an ophthalmologist as either an undifferentiated condition or labeled as retinitis pigmentosa, as was the case in our patient. Without a certain index of suspicion, Refsum disease may be difficult to diagnose. This is evident from a study that reported an average of 11 years between initial symptom onset and when a biochemical diagnosis of Refsum disease was made.^6^ The same study found that development of the neurological manifestations of disease, including peripheral neuropathy and cerebellar ataxia, typically prompted the diagnosis even though the ophthalmic features were present at an earlier stage.^6^ This highlights the importance of regular ophthalmic follow‐up for patients with retinal dystrophies as nonocular signs may be initially subtle or even present at a later stage. In addition, our case emphasizes the importance of molecular genetic analysis in patients with inherited retinal dystrophies. This approach increases the likelihood of detecting syndromic forms of retinitis pigmentosa and allows for earlier subspecialty referral and possible intervention to reduce disease morbidity.

Despite several studies suggesting that retinal degeneration can be slowed in patients with Refsum disease given appropriate phytanic acid restriction, there have been no reports thus far demonstrating improved retinal function.^1^, ^3^ One possible reason for this is that the onset of retinopathy often occurs a number of years before a diagnosis of Refsum disease is made. As a result, the degree of retinal degeneration is usually severe at the time of diagnosis and this can limit the potential for retinal recovery with dietary intervention.^6^, ^7^ Another possible explanation for a lack of demonstrated improvement is that methods of vision assessment have not been fully standardized for these patients, given the rarity of this condition, making any true retinal recovery difficult to ascertain. For example, one study described preserved visual acuities in a significant number of patients following dietary intervention leading to decrease in phytanic acid level.^6^ A portion of these patients, however, had also undergone cataract extraction during the therapeutic interval, thus confounding a true comparison of visual acuity before and after dietary intervention. Despite the presence of an underlying retinal dystrophy, visual acuity has still been shown to improve following cataract extraction in these patients.^8^

Even though our patient had extensive retinal degeneration at the time of diagnosis, his subjective impression of improved vision following dietary intervention prompted us to repeat the flicker ERG using the same ISCEV standards as the initial test. We identified increased amplitudes of the 30 Hz flicker ERG waveforms in both eyes, supporting his own perception of improvement, despite a lack of measured improvement in Snellen visual acuity. The 30 Hz flicker ERG is sensitive to residual cone photoreceptor activity and has been used to assess remaining visual function in patients with extinguished ERGs and advanced vision loss.^9^ The increase in flicker ERG amplitudes demonstrated in our case is substantial and therefore does not likely represent background signal or noise. One hypothesis to explain this improvement despite no change evident in visual acuity is improved speed of neurotransmission in the remaining outer retinal neurons. In support of this proposal, a previous study demonstrated enhanced conduction velocity in motor neurons following lipid apheresis in patients with Refsum disease.^10^ To the best of our knowledge, this is the first report of improved retinal function, demonstrated by ERG testing, in a patient with Refsum disease following dietary intervention. In our case, retinal recovery was small and evident on ERG testing only.

Although our patient was able to achieve a nearly 40% reduction in serum phytanic acid from 449 μmol/L to 273 μmol/L following 4 months of dietary intervention, he had difficulty maintaining this compliance. As a result, his third serum phytanic acid level was 396 μmol/L 3 months later. A study examining the effectiveness of at least 10 years of dietary intervention noted that 80% of patients were able to achieve serum phytanic acid levels of <300 μmol/L.^11^ The same study found that patient noncompliance with dietary suggestions was not uncommon (33%) but was typically only intermittent (lasting less than 1 year).^11^ Our patient still demonstrated an overall reduction in serum phytanic acid levels but longer follow‐up with stricter compliance will be required to reduce his levels further.

Limitations of our study include poor diet recall and the absence of 3‐day food records from our patient. Thus, it was not possible for us to accurately quantify and calculate phytanic acid intake. Our patient reported, however, strict adherence to the dietary recommendations prior to the second serum phytanic acid level and admitted to noncompliance prior to the third serum level. This was consistent with the changes evident in his serum phytanic acid levels. Given this, we believe that the reduction seen in serum phytanic acid is likely due to decreased phytanic acid intake following dietary intervention. Due to the above limitations, however, this cannot be confirmed with certainty.

In conclusion, our results provide evidence for the hypothesis that some functional retinal recovery may, indeed, be possible in patients with Refsum disease. Just as symptoms of ichthyosis, peripheral neuropathy, and ataxia can improve with dietary intervention, it logically follows that the potential for visual function to improve may exist as well, especially if treatment is initiated early in the disease course and before significant retinal degeneration has occurred. Finally, our finding of improved ERG responses supports incorporating electroretinography as a standardized and objective assessment of treatment response in patients with Refsum disease.

## CONFLICT OF INTEREST

The authors declare that they have no conflict of interest.

## INFORMED CONSENT

All procedures followed were in accordance with the ethical standards of the responsible committee on human experimentation (institutional and national) and with the Helsinki Declaration of 1975, as revised in 2000. Informed consent was obtained from all patients for being included in the study.

## ANIMAL RIGHTS

This article does not contain any studies with animal subjects performed by the any of the authors.

## Supporting information


**Supplementary Table S1** A table of dietary recommendations created by our center to facilitate achieving and maintaining low phytanic acid intake in patients with Refsum disease.
