# Supplementary material for: Improved electroretinographic responses following dietary intervention in a patient with Refsum disease
Source: JIMD Rep. 2020 Jul 12;55(1):32–7. doi: 10.1002/jmd2.12147 (PMC7463047; doi:10.1002/jmd2.12147)
Supplement: Supplementary file 1 — Supplementary Table S1 A table of dietary recommendations created by our center to facilitate achieving and maintaining low phytanic acid intake in patients with Refsum disease. [file JMD2-55-32-s001.doc]

**REFSUM SYNDROME**

**Low Phytanic Acid Diet:** found in foods obtained from animals which live mainly on green plants

| **Food Group** | **Foods not Allowed** | **Foods Permitted** |
| --- | --- | --- |
|  |  |  |
| **Meat** | Beef, beef stock, lamb, mutton, venison, goose, rabbit, organ meats from all animals (liver, thymus, kidney). | Pork, ham, bacon, chicken, duck, turkey, vegetarian meat substitutes (soya products). |
|  |  |  |
| **Fish** | All Fish (including shellfish and fish oils) except those noted. | Canned tuna in water (once weekly), small portions of low fat white fish like cod and haddock. |
|  |  |  |
| **Dairy** | All high fat dairy products including, cow’s milk or yogurt with 1% or more fat, cream (including cream soups, cream sauces, and drinks made with cream), condensed milk, evaporated milk, goat or sheep milk, traditional cheeses (even light), processed cheese, cheese spreads, dairy desserts such as rice pudding or ice cream. | Fat free dairy products such as skim milk, fat-free yogurt or cheese, soya milk, coffee mate, soya or vegan cheeses or ice creams. |
|  |  |  |
| **Eggs/Nuts** | Goose eggs, peanuts, walnuts. | Hen’s eggs, all nuts (except peanuts/walnuts). |
|  |  |  |
| **Bread/Cereals** | Commercial baked products such as muffins, cupcakes, pastries, cake, croissants or donuts (contain butter). | All cereals (wheat, oats, rice, maize, sago, tapioca, corn), all breakfast cereals, pasta, bread, flour, desserts made with margarine. |
|  |  |  |
| **Fruit/Vegetables** | None. | All allowed. |
|  |  |  |
| **Oils/Fats** | Butter (including spreads made with butter or animal fats), suet, soya oil, peanut oil. | Vegetable oils (groundnut, corn, olive, canola, safflower, sunflower), hydrogenated oils, lard. |
|  |  |  |
| **Sweets** | Milk chocolate, plain chocolate containing butterfat. | Chocolate containing no milk or butterfat (plain chocolate with soya), sweets containing no fat (jellies, boiled sweets, liquorice). |
